# Hysteresis Compensation in Force/Torque Sensors Using Time Series Information

**DOI:** 10.3390/s19194259

**Published:** 2019-09-30

**Authors:** Ryuichiro Koike, Sho Sakaino, Toshiaki Tsuji

**Affiliations:** 1 Graduate School of Science and Engineering, Saitama University, Sakura-ku, Saitama City, Saitama 338-8570, Japan; 2 Department of Intelligent Interaction Technologies, University of Tsukuba, Tsukuba, Ibaraki 305-8577, Japan

**Keywords:** force sensing, force sensor, tactile sensing, linear regression, hysteresis compensation

## Abstract

The purpose of this study is to compensate for the hysteresis in a six-axis force sensor using signal processing, thereby achieving high-precision force sensing. Although mathematical models of hysteresis exist, many of these are one-axis models and the modeling is difficult if they are expanded to multiple axes. Therefore, this study attempts to resolve this problem through machine learning. Since hysteresis is dependent on the previous history, this study investigates the effect of using time series information in machine learning. Experimental results indicate that the performance is improved by including time series information in the linear regression process generally utilized to calibrate six-axis force sensors.

## 1. Introduction

Force control has long been a fundamental technology for robotics and is still being actively studied. Known techniques include using an external force observer [1] or series elastic actuators [2], while the most common approach utilizes force sensors. Force sensors enable the acquisition of force information in a simple manner. However, they typically face problems of hysteresis, where measurements are affected by previously applied loads. Force sensors acquire the deformation volume of a strain body using sensing elements (e.g., strain gauges [3,4,5], capacitance sensors [6,7], resistive sensors [8] or optical sensors [9,10,11]) and estimate the force applied to the deformation volume. Most of these are designed such that the deformation volume of the strain body and the force have a linear relationship, and this relationship is often estimated through linear regression. However, nonlinearity results from the physical properties of the strain body and the attachment of sensing elements. Several studies have been conducted to determine the relationship between the values obtained by the sensors on a strain body and the applied force, to discuss this nonlinearity. Yingkun et al. proposed a hybrid calibration approach combining linear regression and a support vector machine (SVM) (a form of machine learning), which improved the performance compared with calibration solely using linear regression [12]. Li et al. utilized an SVM application called support vector regression (SVR) to perform regression for calibration [13]. In addition, some previous studies have performed calibration solely using a neural network (NN) without linear regression [14,15,16]. Oh et al. increased the number of NN layers, with the intent of further improving the performance [17]. In general, as the number of NN layers is increased a larger amount of data is required for learning. The calibration of force sensors often involves the use of calibration devices [18]. Thus, it is not possible to prepare a large amount of data as these are operated manually.

As stated above, many studies have considered the calibration of force sensors, and, although techniques using machine learning have been proposed, these do not solve the problem of hysteresis. An example of a study addressing hysteresis is that of Tomo et al. [19]. The authors developed a three-axis skin sensor using Hall elements. However, the evaluation of its performance revealed no compensation of hysteresis. Urban et al. compensated for hysteresis using a two-axis skin sensor [20] and Horii et al. utilized a three-axis tactile sensor [21]. These were calibrated using machine learning but have not been expanded to six axes. Therefore, the purpose of this study is to perform calibration of a six-axis force sensor using machine learning and compensate for the hysteresis phenomenon. Creep also occurs when an object deforms through the continuous application of a constant load and is particularly pronounced for materials such as resins. The main difference between creep and hysteresis is that the former is dependent on both time and the previous state, while the latter depends only on the previous state. As a method to compensate for the creep phenomenon was proposed in Reference [22], and as hysteresis is a more critical issue, this study focuses on hysteresis compensation. Accounting for the fact that hysteresis is only dependent on the previous history, the technique proposed in this study compensates for errors by keeping past data on hand. Although conventional machine learning methods also show a similar effect, the method in this paper has an advantage that the hysteresis is reduced with a simple learning method with small training data.

This paper is organized as follows. In Section 2, we describe force sensors and the basic principles behind machine learning. Next, we explain the proposed technique in Section 3, and describe our experiment in Section 4. Finally, we present our conclusions in Section 5.

## 2. Basic Principles

### 2.1. Signals of a Strain Gauge-Type Force Sensor

Six-axis force sensors, measuring the three-axis directional force and three-axis moment, are the most commonly used force sensors for robots. This study utilizes the strain gauge type, while the proposed method could be utilized for other types as long as the flexure element is subject to the hysteresis or creep phenomena. Strain measurements need to be acquired at a minimum of six locations. Assuming that the strain S is measured in i≥6 locations in a strain body and the load applied to the strain body is L, S and L are expressed as follows:

(1) $$\begin{array}{ccc} L & = & {\left[F_{x},F_{y},F_{z},M_{x},M_{y},M_{z}\right]}^{⊤} \end{array}$$

(2) $$\begin{array}{ccc} S & = & {\left[S_{1},S_{2},\cdots ,S_{i}\right]}^{⊤} \end{array}$$

where F[N] and M[Nm] represent the force and moment produced at the sensor center *O*, respectively, and the subscripts x,y, and *z* represent the coordinates in the Cartesian coordinate system. In other words, practically speaking the acquisition of the strain S using a strain gauge and the subsequent conversion of this value to L via the function f(·) play the role of a force sensor. Here, f(·) is a function expressing the relationship between the strain S and applied load L and this function should be estimated from the results of preliminary experiments.

### 2.2. Linear Regression

Supposing that S and L are linearly related, the relation can be expressed as follows according to an i×6 calibration matrix C:

(3) $$\begin{array}{c} S=CL \end{array}$$

Furthermore, when the inverse or pseudo-inverse matrix of the calibration matrix C is utilized, we have

(4) $$\begin{array}{ccc} L & = & C^{-1}S\;\;\;\;\left(i=6\right) \end{array}$$

(5) $$\begin{array}{ccc} L & = & {\left(C^{T}C\right)}^{-1}C^{T}S\;\;\;\;\left(i>6\right) \end{array}$$

As Equation (5) shows, the applied load L can be estimated from the strain output S acquired with the strain gauge.

Next, we describe how to determine the calibration matrix. Here, the set consisting of the applied load L and strain S at a certain time is already known and the number of samples is *n*. In other words, $L=[L^{1},L^{2},\cdots ,L^{n}]$ and $S=[S^{1},S^{2},\cdots ,S^{n}]$ have already been provided. Here, $L^{k}={\left[F_{x}^{k},F_{y}^{k},F_{z}^{k},M_{x}^{k},M_{y}^{k},M_{z}^{k}\right]}^{⊤}$ and $S^{k}={\left[S_{1}^{k},S_{2}^{k},\cdots ,S_{i}^{k}\right]}^{⊤}$. Hereinafter, for the sake of simplicity we shall only consider $F_{x}$ and discuss the case with i=6. Assuming the inverse matrix $C^{-1}$ of the calibration matrix C to be

(6) $$\begin{array}{c} C^{-1}=\left[\begin{array}{cccc} a_{11} & a_{12} & \ldots & a_{16}\\ a_{21} & a_{22} & \ldots & a_{26}\\ \vdots & \vdots & \ddots & \vdots \\ a_{61} & a_{62} & \ldots & a_{66} \end{array}\right] \end{array}$$

the predicted value $F_{x}^{k*}$ of the *k*th sample $F_{x}^{k}$ is expressed as

(7) $$\begin{array}{c} F_{x}^{k*}=a_{11}^{k}*S_{1}^{k}+a_{12}^{k}*S_{2}^{k}+\cdots +a_{16}^{k}*S_{6}^{k} \end{array}$$

Then, to ensure that the residual sum of squares of the actual applied load and predicted value

(8) $$\begin{array}{c} \sum_{k=0}^{n}d_{k}^{2}=\sum_{k=0}^{n}{\left(F_{x}^{k}-F_{x}^{k*}\right)}^{2} \end{array}$$

is minimized, the constants $\left[a_{11},a_{12},\ldots ,a_{16}\right]$ are derived using the least-squares method, making it possible to determine the inverse matrix $C^{-1}$ of the calibration matrix C. Other rows are also calculated in the same token.

## 3. Proposed Method

### 3.1. Machine Learning Incorporating Time Series Information

#### 3.1.1. Acquiring Time Series Information

**Figure 1:**
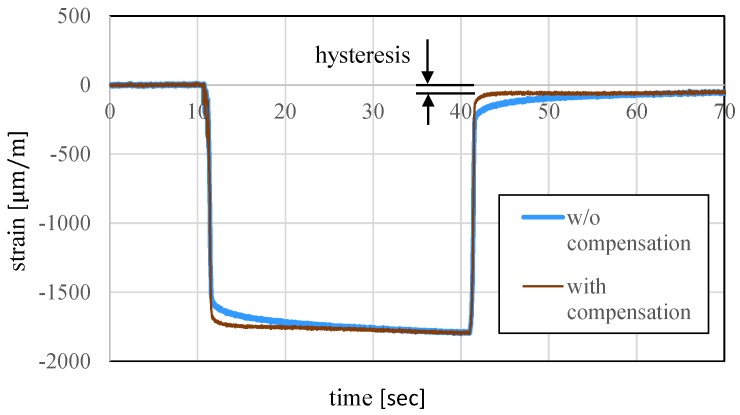
One-axis hysteresis and creep phenomena.

**Figure 2:**
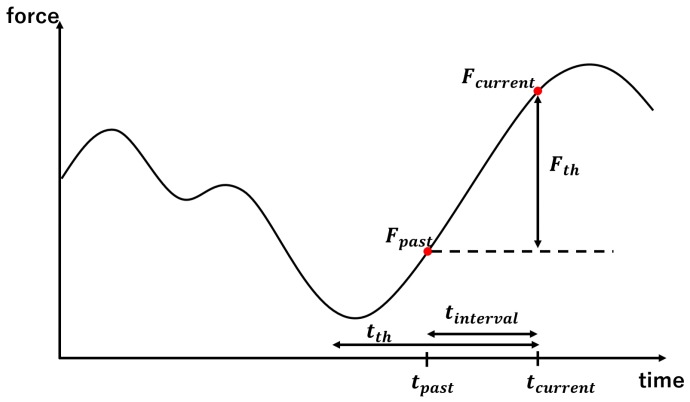
Conceptual diagram for utilizing time series information.

The hysteresis and creep phenomena occur more prominently in resin force sensors than metal force sensors. Even if the sensor is composed of metal, hysteresis is the largest source of error and its compensation is an important issue for a stronger performance. To this end, the consideration of time series information appears to be effective. Figure 1 illustrates the strain behavior when a 10 kg weight is placed on top of a force sensor and later replaced. The result shows how the errors occur owing to the hysteresis and creep phenomena. In our previous study, we attempted to utilize regression with past data with a constant interval time [23,24] and finally concluded that it is difficult to determine the time interval, because creep and hysteresis exhibit different features. Reference [22] has shown that over 80% of the creep error can be cancelled by a single-order compensation. This study therefore focuses on hysteresis compensation. The dark thin line in Figure 1 shows the compensated result. As confirmed in Figure 1, the hysteresis effect remains after this compensation and is the most critical issue for force/torque measurement. To compensate for hysteresis, we focus on times that the force significantly changes and utilize the time series information from such instances. Figure 2 presents a conceptual diagram. In the figure, $F_{\mathrm{th}}$ represents the threshold value of the force and $F_{\mathrm{current}}$ and $F_{\mathrm{past}}$ represent the current force and first force to exceed the threshold value retrospective of the current value, respectively. As the time series information actually employed consists of the strain S, this variable should be specifically explained. We determine the “present” and “time at which the force changes to the threshold value or higher for the first time retrospectively from the present,” use these as $t_{\mathrm{current}}$ and $t_{\mathrm{past}}$ and denote the difference as $t_{\mathrm{interval}}$. In other words, the following equation holds:

(9) $$\begin{array}{c} t_{\mathrm{past}}=t_{\mathrm{current}}-t_{\mathrm{interval}} \end{array}$$

Here, when the strain at $t_{\mathrm{past}}$ is $S_{\mathrm{past}}$ and that at $t_{\mathrm{current}}$ is $S_{\mathrm{current}}$, the current force is expressed by

(10) $$\begin{array}{c} F_{\mathrm{current}}=f\left(S_{\mathrm{past}},S_{\mathrm{current}}\right) \end{array}$$

Here, f(·) is a function expressing the relationship between the current and past strains and the current force. We express the function f(·) through machine learning. In this manner, we can variably handle past data from any time and not just a specific earlier time.

However, two problems emerge with this technique. The first is how to proceed when the force never retrospectively changes to the threshold value or higher. In this situation, we decided to employ the current data as the past data. In other words,

(11) $$\begin{array}{c} F_{\mathrm{current}}=f\left(S_{\mathrm{current}},S_{\mathrm{current}}\right) \end{array}$$

The hysteresis and creep phenomena occur to a considerable extent when the force changes to a high degree but hardly occur when the change in the force is small. Thus, it is expected that if the change in the force is small a conventional technique using only the current values should be sufficient.

According to this process, if the change in the force is small then only the current values are utilized for force estimation. The second problem is that when retrospectively searching for changes in the force at the threshold value or higher, sometimes the search goes all the way back to the first datum in a dataset. For example, if the changes in the force following a single large change at the threshold value or higher are small. When retrospectively searching for large changes in the force under such circumstances, previous data are utilized as past data to the extent that they barely affect the current value. This is undesirable in practice. Therefore, we determined an upper limit for retrospective searches with this technique. In other words, if searching retrospectively from the present to the threshold time value $t_{\mathrm{th}}$ identifies no changes in force exceeding the threshold value, then the above process is performed. To summarize the above, the values used as past data $S_{\mathrm{past}}$ for the current data $S_{\mathrm{current}}$ are expressed as follows:

(12) $$\begin{array}{c} S_{\mathrm{past}}=\left\{\begin{array}{ll} S_{\mathrm{past}} & \left(t_{\mathrm{interval}}\leq t_{\mathrm{th}}\right)\\ S_{\mathrm{current}} & \left(t_{\mathrm{interval}}>t_{\mathrm{th}}\right) \end{array}\right. \end{array}$$

Performing these processes at each time point results in the acquisition of time series information. Such an algorithm is implemented in our method and it is confirmed that the required calculation is sufficiently small for real-time processing in the control systems of robots.

#### 3.1.2. Utilization of Time Series Information for Machine Learning

**Figure 3:**
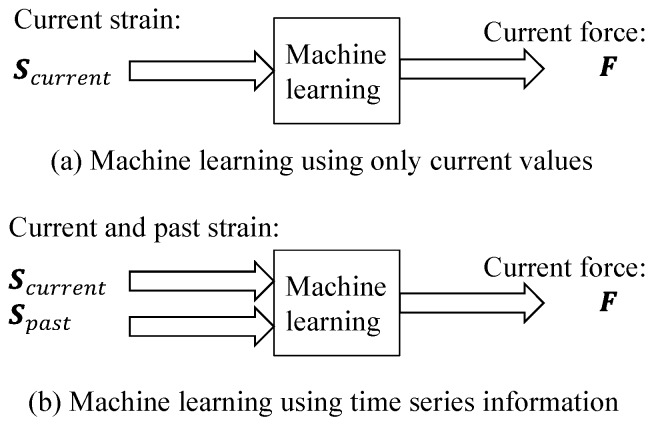
Machine learning inputs and outputs in this study.

The machine learning approach considering time series information is illustrated in Figure 3. Linear regression, an NN and a hybrid model described in the next subsection are the candidate machine learning methods. The strain S and applied load L are provided as the input and output, respectively, for training data during training. During testing, when the strain S is entered for the input the machine learning process predicts the applied load L. When using time series information, the input strain S is $S=[S_{\mathrm{current}},S_{\mathrm{past}}]$. Here, $S_{\mathrm{current}}$ and $S_{\mathrm{past}}$ are the current and past strains, respectively.

### 3.2. NN and Hybrid Model

**Figure 4:**
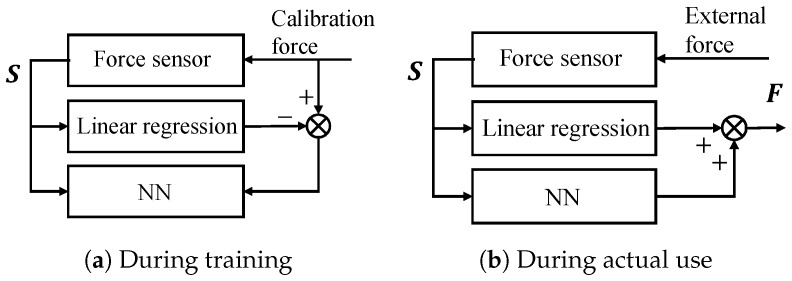
Hybrid model configuration.

This study mainly utilizes linear regression, while two conventional machine learning methods are also introduced as candidates to apply our proposal. The first is an NN [15]. A commonly employed feedforward structure is introduced. In addition, the hybrid model in Reference [12] is extended to an NN. Figure 4 illustrates the hybrid model of linear regression and an NN. During training, the applied load L and strain S at a certain time are provided as training data to determine the actual relationship. By applying linear regression to these training data, it is possible to approximate the rough relationship between L and S. The system learns the differences therein and the relationship with the strain S using the NN. In other words, if the linear function is $f_{1}\left(\cdot \right)$, then the output $L-f_{1}\left(S\right)$ is learned when inputting the strain S into the NN.

## 4. Experiment

### 4.1. Experimental Equipment

#### 4.1.1. Strain Gauge and Strain Measuring Device

**Figure 5:**
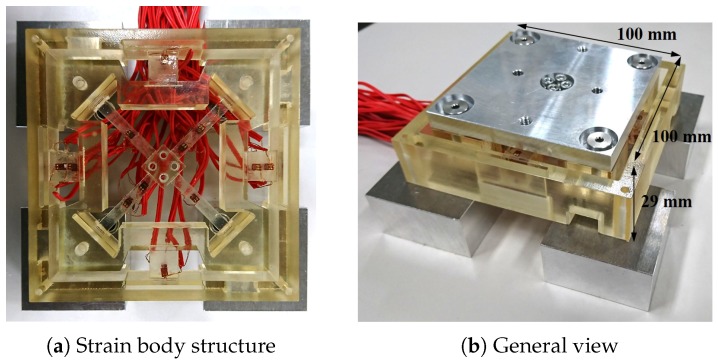
Resin high-dynamic-range force sensor.

Figure 5 illustrates the utilized force sensor. The sensor is composed of a Keyence Corporation AGILISTA-3100 3D printer and AR-M2 modeling material [22]. AR-M2 is a resin Material, with almost the same physical properties as ABS resin. The rated force of the flexure element is 12 N and the performance was evaluated with external force smaller than the rated force. By using resin material instead of metal, it is possible to produce a lightweight and inexpensive force sensor. Because the main issue of force sensors with resin material is the error caused by hysteresis, this sensor is a good candidate to verify the proposed method.

#### 4.1.2. Robot Arm-Based Force Application Device

**Figure 6:**
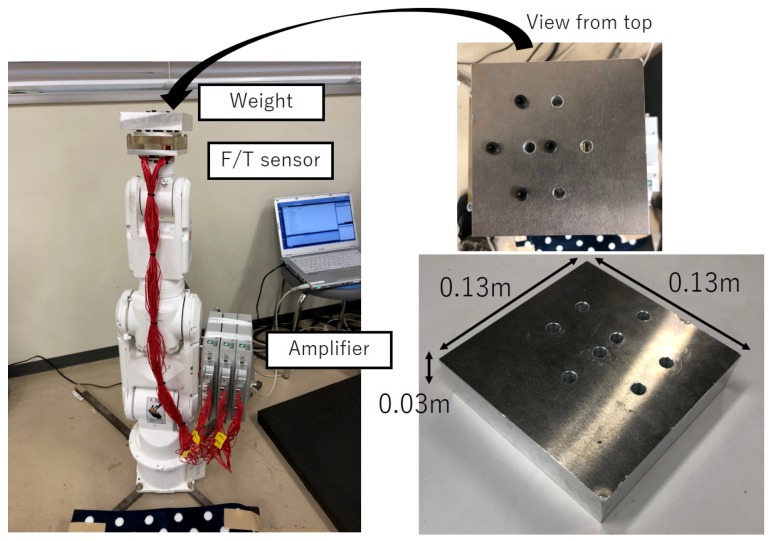
General view of the experimental device.

Figure 6 illustrates the force application device used for data collection. This device comprises a robot arm, force sensor, weight and strain gauge, with a strain measuring device installed within the force sensor. As the robot arm, we utilized a “MOTOMAN-MH3F” by the Yaskawa Electric Corporation. The weight for calibration was a rectangular aluminum block, with dimensions of 0.13 m × 0.13 m × 0.03 m. In addition, the holes for fixing are open at positions in the center and offset from the center by 0.00, 0.02 and 0.015 m. If the fixing is at the center hole, then no moment is applied and it is only possible to measure a three-axis force. If the fixing is at an offset position hole, then it is possible to create a moment, enabling six-axis measurement. The weight for calibration has a mass of 1.128 kg. The size of the load can be adjusted by changing the number of weights. Using these values, we computed the applied load L in an absolute coordinate system from the positioning of the robot. Sampling time of the F/T sensor was 50 ms.

### 4.2. Learning

**Figure 7:**
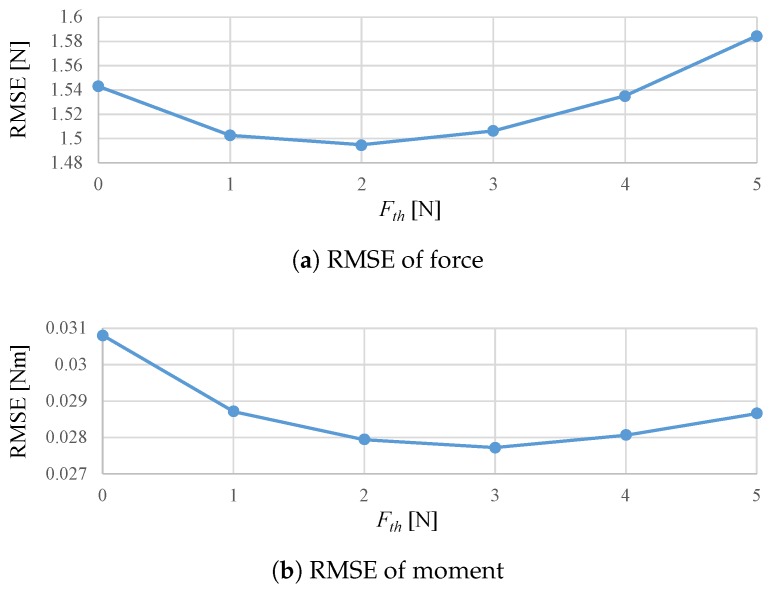
Pre-examination to determine $F_{th}$.

**Figure 8:**
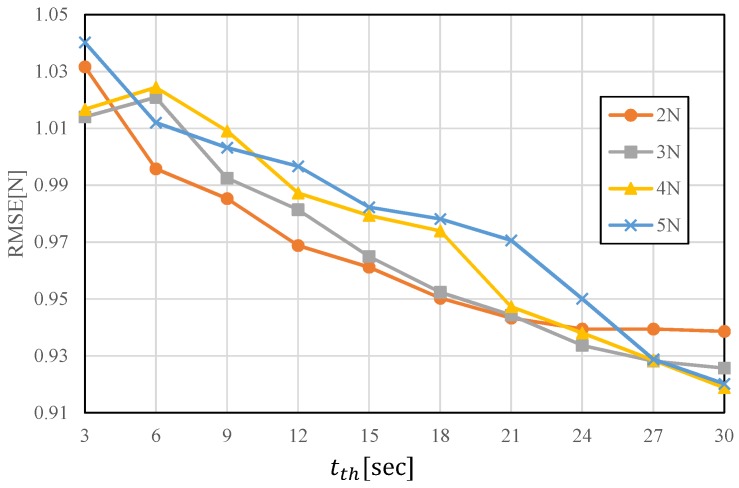
Pre-examination to determine $t_{th}$.

This subsection describes the regression of the applied load acquired in the previous section and its strain values using linear regression, an NN and the hybrid model in detail. We look back at past data from the current data using each technique and then utilize the data from the time at which the force first exceeded the threshold $F_{th}$. We measured the average hysteresis value after rated output load was applied and the result was 2.64 N. Therefore, the threshold value $F_{th}$ was set at 3 N, around the average hysteresis. Figure 7 shows the root mean squared error (RMSE) with different $F_{th}$. Note that the results with $F_{th}=0$ are equivalent to conventional LR methods without time series information. The error was the smallest when $F_{th}$ was around 2 to 3 N and these results support the validity of above-mentioned threshold setting policy. Figure 8, the results of the preliminary test with different $t_{th}$, show that the RMSE becomes smaller with longer time threshold $t_{th}$, while longer $t_{th}$ ends up with longer calculation time. We set $t_{th}$ as 15 s considering the trade-off.

#### 4.2.1. NN Structure and the Hybrid Model

A feed-forward structure with three hidden layers was introduced in this experiment as a typical NN. The input layer has a number of nodes matching the number of dimensions (six for only current values, 12 if including time series information) of the strain S and the output layer has six nodes corresponding to the dimensions of applied load L. In addition, the hidden layers each have 100 nodes. The batch size was 100 and the number of epochs was 1000. Furthermore, the loss function was set as the sum of the squared errors of the force/torque responses of the six axes. The general structure of the NN in the hybrid model is the same as that described in the previous subsection.

#### 4.2.2. Training Patterns

The NN performance varies depending on the amount of training data. The performance generally improves as more data are learned. We verified how the performances of linear regression, the NN and the hybrid model varied depending on the amount of training data.

Pattern A (amount of training data: large)
  Six different paths are given in the experiments, so that most of the operating range is covered. The trajectories are all based on sinusoidal waves with random amplitude in each direction. The frequency of the sinusoidal wave was set to 0.05 Hz to avoid error owing to inertia force error. Five of these were used for training and the other was used for testing. Each path was examined with three trajectories with different velocities. Depending on the speed of each trajectory, the recorded time differs as 95 s, 135 s, and 175 s for fast, medium and slow speeds, respectively. We acquired five paths for each of these, recorded them at a rate of 20 samples/s and performed training with a total of 202,500 samples.
Pattern B (amount of training data: small)
  The amount of data used for the training and testing of pattern A was reduced to 1/10th of the original size, simply by decimating the same trajectory. Through this operation, we created a state in which the amount of data was simply reduced and verified the precision of each technique. Depending on the speed of each trajectory, the time recorded differs as 95 s, 135 s and 175 s for fast, medium and slow speeds. We acquired five paths for each of these, recorded them at a rate of 2 samples/s and performed training with a total of 20,250 samples.

### 4.3. Results

#### 4.3.1. Pattern A (Amount of Training Data: Large)

**Figure 9:**
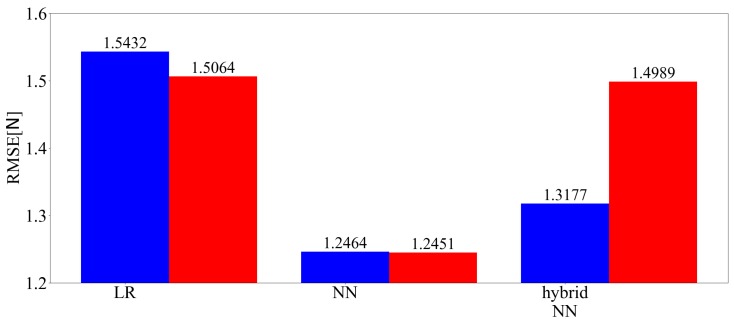
Three-axis root mean square error (RMSE) of the force (learned with 202,500 samples).

**Figure 10:**
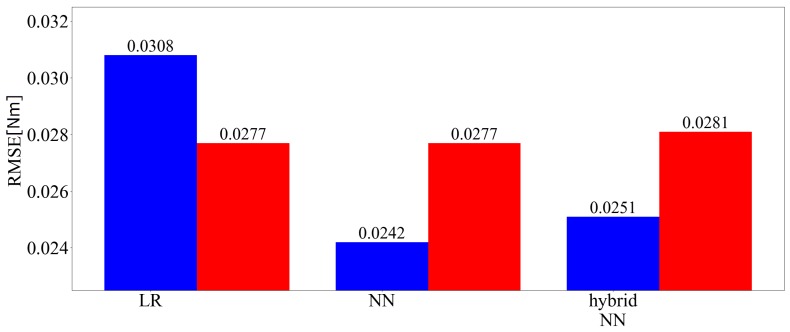
Three-axis RMSE of the moment (learned with 202,500 samples).

**Figure 11:**
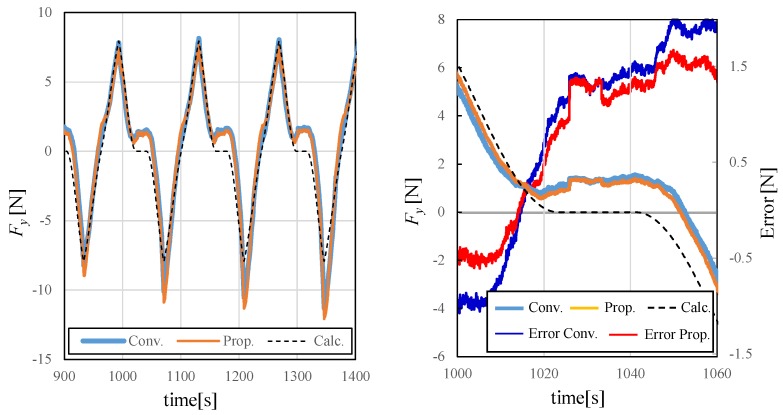
Example of force responses during testing.

Figure 9 shows the three-axis and predicted values of the root mean square error (RMSE) of the force among the applied loads of each technique. In the figure, LR is linear regression, hybrid NN is the hybrid model, blue indicates no time series information and red indicates that time series information is included. Figure 10 shows the three-axis and predicted values of the RMSE of the moment among the applied loads of each technique. Figure 11 shows time responses of an example of a 135 s term trajectory. Here, Conv. denotes the conventional LR method while Prop. denotes the proposed method including time series information. Calc. shows the ideal external force value calculated based on kinetics. Errors of the conventional method and the proposed method are calculated as the difference between the measured value and the calculated ideal value. The errors are shown in the right graph, the enlarged drawing of the left. The responses of the proposed method show that hysteresis is the largest source of the error and the error is reduced by incorporating past information. The responses of the proposed and conventional methods from 1026 s to 1033 s match because the force variation was smaller than $F_{th}$ then.

#### 4.3.2. Pattern B (Amount of Training Data: Small)

**Figure 12:**
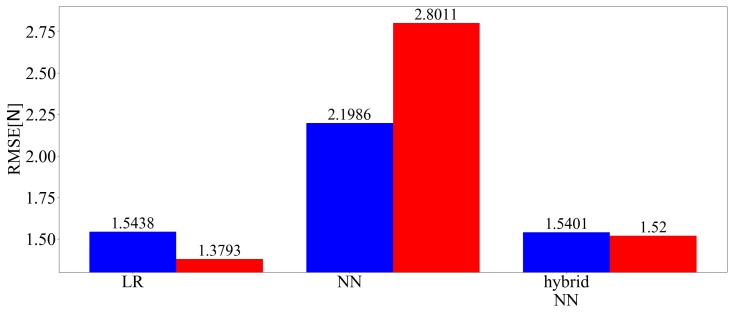
Three-axis RMSE of the force (learned with 20,250 samples).

**Figure 13:**
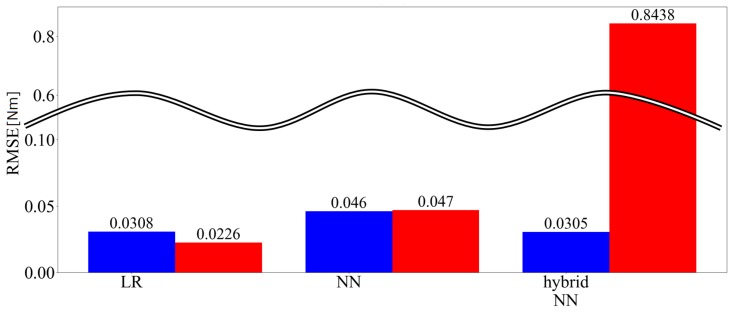
Three-axis RMSE of the moment (learned with 20,250 samples).

Figure 12 shows the three-axis and predicted values of the RMSE of the force among the applied loads of each technique. Figure 13 presents the three-axis predicted value and root-mean-square error (RMSE) of the moment for the applied loads for each technique.

#### 4.3.3. Discussion

When the amount of data for training was high, the inclusion of the time series information reduced the errors in the linear regression, slightly reduced the errors in the NN and increased the errors in the hybrid model. Resin force sensors are likely to be affected by the hysteresis. Therefore, the linear regression output is biased when the force either increases or decreases in the data. By including previous information, if the previous values are smaller than the present values then the proposed method corrects the force to be larger and vice versa. For the NN, as learning was performed when the force became both larger and smaller using only the current values, there was no significant change, even when past information was entered. In the hybrid model, although NN learning sections exist these did not fit well with the linear regression and the present strain output is stronger in nonlinearity than linearity. Thus, the errors tended to decrease when using only the NN.

When the amount of data was small, the inclusion of the time series information reduced the errors in the linear regression, increased the errors for the NN and increased the errors in the hybrid model. For linear regression, it is inferred that the reason is similar to that when the amount of data is high. In addition, it is also inferred that the estimation precision was lower because the amount of data was insufficient. The number of dimensions of the input doubled when the time series information was included and therefore the insufficient amount of data had an influence. Similar errors with the linear regression appeared as when the amount of data was high and the errors with the linear regression and the applied load could not be learned well with the NN as the amount of data was small.

Comparing the cases in which the amounts of data were high and low, the NN performance was the best for a high amount of data and linear regression and the hybrid model performed well when the amount of data was low. The reason for this appears to be that the NN yielded a generally inferior performance when the amount of data was low, as a large amount of data is required for training.

It was found through the examination that when adding time series information to the linear regression, the precision of estimating the applied load was improved. In addition, it was found that the linear regression precisely estimated the applied load when the amount of data was small. From this, it can be said that linear regression is effective when the amount of data cannot be fully guaranteed. In addition, it is possible to improve the performance of a force sensor with large hysteresis by estimating using linear regression instead of an NN. On the other hand, the NN performance is good when a large amount data can be prepared and sufficient data is available learning. Depending upon the application, estimation using an NN is effective if one wants to determine the performance. However, linear regression accounting for time series may be considered to be effective if we simply want to reduce the effects of hysteresis in a commercial force sensor, which requires shorter calibration efforts.

## 5. Conclusions

In this study, we considered time series information for the strain values converted into force information using a six-axis force sensor, to propose a technique for improving the performance. We then performed learning using three approaches: linear regression, an NN, and a hybrid model. The inclusion of time series information in the linear regression method reduced the errors. The addition of time series information in the NN and the hybrid model increased the errors. When we compared the overall performances for a large amount of data, the applied load was estimated with the most precise calibration using the NN. However, when the amount of data was small the applied load was estimated most precisely by linear regression including time series information. These results support the claim for the advantage of the linear regression method including time series information.
